# Selective Brain Cooling: A New Horizon of Neuroprotection

**DOI:** 10.3389/fneur.2022.873165

**Published:** 2022-06-20

**Authors:** Ji Man Hong, Eun Sil Choi, So Young Park

**Affiliations:** ^1^Department of Neurology, Ajou University School of Medicine, Ajou University Medical Center, Suwon, South Korea; ^2^Department of Biomedical Science, Ajou University School of Medicine, Ajou University Medical Center, Suwon, South Korea

**Keywords:** selective brain cooling, systemic cooling, therapeutic hypothermia, brain temperature, human application, neuroprotection

## Abstract

Therapeutic hypothermia (TH), which prevents irreversible neuronal necrosis and ischemic brain damage, has been proven effective for preventing ischemia-reperfusion injury in post-cardiac arrest syndrome and neonatal encephalopathy in both animal studies and clinical trials. However, lowering the whole-body temperature below 34°C can lead to severe systemic complications such as cardiac, hematologic, immunologic, and metabolic side effects. Although the brain accounts for only 2% of the total body weight, it consumes 20% of the body's total energy at rest and requires a continuous supply of glucose and oxygen to maintain function and structural integrity. As such, theoretically, temperature-controlled selective brain cooling (SBC) may be more beneficial for brain ischemia than systemic pan-ischemia. Various SBC methods have been introduced to selectively cool the brain while minimizing systemic TH-related complications. However, technical setbacks of conventional SBCs, such as insufficient cooling power and relatively expensive coolant and/or irritating effects on skin or mucosal interfaces, limit its application to various clinical settings. This review aimed to integrate current literature on SBC modalities with promising therapeutic potential. Further, future directions were discussed by exploring studies on interesting coping skills in response to environmental or stress-induced hyperthermia among wild animals, including mammals and birds.

## Introduction

Acute brain damage, including initial primary injury from acute stroke, traumatic brain injury, post-cardiac arrest brain syndrome, and brain tumor, shares common secondary brain injury that occurs in hours to weeks, including systemic complications and destructive cellular cascades (1–3). Animal experiments and clinical trials have confirmed that therapeutic hypothermia (TH) for acute brain damage that prevents irreversible cell death in the brain is beneficial for post-cardiac arrest syndrome and neonatal hypoxic encephalopathy by avoiding ischemia-reperfusion injury (4–6). Results from previous studies and experiments have shown that various cells in the body have a lower metabolic rate and a cell protection effect with the decrease in body temperature (7). Although most clinical studies have shown that the temperature range associated with better outcomes is 32–35°C, it has been suggested that appropriate management of adverse events following systemic hypothermia is indispensable for successful clinical benefits (8). Current clinical practice mainly involves whole-body cooling (9), but lowering the systemic temperature to below 34°C can lead to severe complications (10, 11). Therefore, selective brain cooling (SBC) can be a good alternative to reducing serious systemic complications and improving protection against acute brain damage in conditions where the basal metabolic rate is higher than that in other organs, which can easily cause fatal cerebral damage (12, 13).

Although the brain accounts for only 2% of the total body weight, it consumes 20% of the body's total energy and is required a continuous supply of glucose and oxygen to maintain function and structural integrity (7). Such a physiological vulnerability of the brain can be more strikingly obvious in patients with acute brain damage such as post-cardiac arrest syndrome and/or neonatal hypoxic encephalopathy with the pathophysiology of ischemia-reperfusion (14). SBC is defined as the lowering of the brain temperature either locally or below arterial blood temperature. Theoretically, temperature-controlled SBC can be more beneficial than systemic cooling for human applications (15). Various SBC methods have been introduced to selectively cool the brain to minimize systemic TH-related adverse events (12). However, technical setbacks of current SBCs, such as insufficient cooling power and relatively expensive coolant and/or irritating effects on skin or mucosal interfaces, limit its application (15).

This review will provide an in-depth systemic literature review of SBC, its mechanism of action, and therapeutic opportunities and future directions based on various coping mechanisms in animals.

## Limitations of Systemic Therapeutic Hypothermia

TH is a common term for defining intentional cooling in core body temperature and has evolved over decades into a strategy for a more comprehensive control of body temperature. It is now called targeted temperature management (TTM) (11, 16, 17). Clinical trials have proven that TH is effective in post-cardiac arrest and neonatal hypoxic-ischemic encephalopathy (18–20). Recent guidelines have stipulated that TTM is suitable for comatose patients who remain unresponsive after the successful return of spontaneous circulation (21). However, such positive findings are mainly based on systemic TTM for the core body temperature (22).

The neuroprotective effect of cooling is explained by the pleiotropic mechanism of TH against ischemic damage (7, 23). The proven mechanisms of TH that create ischemic tolerance are explained by (1) metabolic depression; (2) reduced oxygen and energy needs of the cell, (3) anti-excitotoxicity, anti-inflammatory, anti-blood mechanisms; and (4) inhibition of blood-brain barrier breakdown (8). Systemic cooling experiments in various ischemic models of rodents, pigs, and rabbits (24, 25) have shown that TH has neuroprotective benefits with respect to reducing infarction size and improving neuro-behavioral performance (e.g., neurologic deficit score, cerebral performance category, and overall performance category) without increasing the risk of serious complications (24, 25). Systemic TH (core body temperature, 33–35°C) has been shown to be beneficial not only for neonates with ischemic encephalopathy but also for comatose adult out-of-hospital cardiac arrest patients with both shockable and non-shockable rhythms (26–29). In addition, cardiac surgery has been routinely performed with extracorporeal blood cooling or controlled cooling, causing profound hypothermia by reducing the core body temperature to ≤ 25°C, to protect the brain from ischemic damage during circulatory arrest or vascular clamping (30).

**Figure 1:**
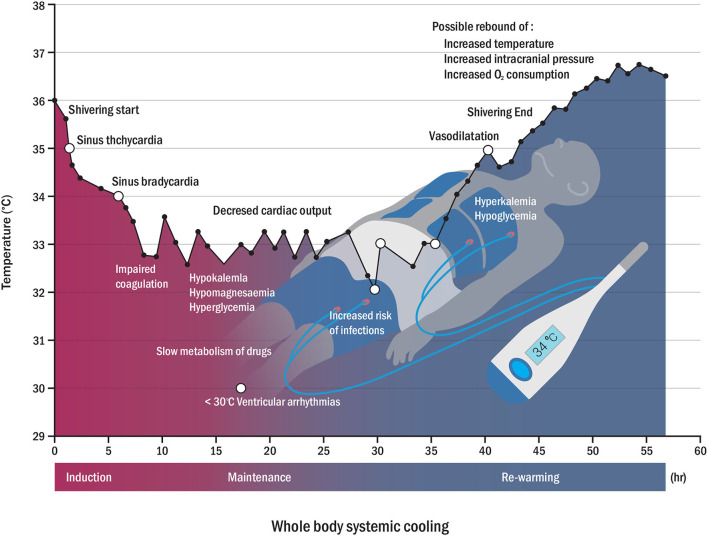
Physiological changes (open circle) and possible side effects (closed circle) according to temperature variation during whole-body cooling. Possible complications may appear in reverse patterns of rewarming complications (hyperkalemia, hypoglycemia, and rebound of increased intracranial pressure, among others) (7, 34).

Acute brain damage may lead to distal organ damage even in the absence of systemic disease or inflammation (31). Since this type of injury affects not only the brain but also the whole body, a holistic therapeutic approach may be required (32, 33). Although systemic TH can help alleviate this type of brain damage, it can cause systemic complications that affect biological survival processes. Induction and maintenance of systemic hypothermia in homeothermic mammals causes physiological side effects, and some side effects may lead to morbidity and death (10). The side effects of TH can be systemically classified into cardiac, hematologic, immunologic, and metabolic complications (10, 34). Common adverse events of TH may include hyperglycemia, shivering, bradycardia, electrolyte abnormalities, acute kidney injury, pneumonia, and hypercoagulability or hypocoagulability syndrome (35). Physiological adverse events due to systemic TH require specialized intensive care resources, sedatives and muscle relaxants, mechanical ventilators, and their combinations (36, 37). Thus, these physiological complications should be carefully considered and closely monitored in the intensive care unit, and patients considering TH should be admitted to the intensive care unit (38). This requirement could be another obstacle to the application of systemic TH. Figure 1 shows examples of complications during TH. Recently, drug-induced hypothermia has gained interest as an alternative option to avoid the complications of systemic TH (39). It has been reported that thermoreceptor-targeted drugs may be an effective strategy for stroke treatment in conscious subjects, even when initiated after a significant period of time following reperfusion (40). Combination therapy of pharmacological and physical TH methods may effectively reduce side effects by decreasing the drug dosage and the time required to reach the therapeutic target (40, 41). Thus, we believe that SBC and drug-induced TH can be a promising new combination therapy option for neuroprotection.

**Figure 2:**
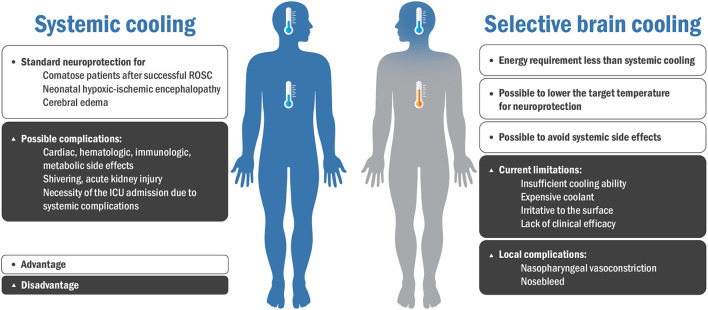
Advantages and disadvantages of systemic cooling and selective brain cooling. ROSC, recovery of spontaneous circulation; ICU, intensive care unit.

High-quality TTM protocols have been recently proposed to minimize the side effects of systemic cooling and maximize efficiency (42). However, systemic cooling still has many drawbacks and requires a new approach without compromising conventional systemic cooling methods (42, 43). In this context, SBC can be a promising neuroprotection approach to avoid the adverse effects of systemic TH (12, 44). Figure 2 shows the advantages and disadvantages of systemic cooling and SBC.

## Clinical Feasibility of Selective Brain Cooling

SBC has been developed over the past decades, and it is an alternative approach that avoids the adverse effects of systemic cooling. In SBC, the brain is selectively cooled to achieve neuroprotection while the core body temperature is maintained within the normal range (45). As such, it may achieve the target temperature of the brain faster and in a cost-effective manner, compared to systemic cooling (45, 46). SBC can be conveniently performed in hospitalized patients as well as in emergency deployment using a compact SBC device. SBC does not require sedatives and paralytics to prevent discomfort and shivering at temperatures below 35°C, as is the case with systemic cooling. The intranasal cooling method, one of the SBCs, can be applied even during the arrest of a patient's blood circulation (47). Furthermore, side effects of systemic cooling such as pneumonia, venous thrombosis, and hypotension may be significantly mitigated in SBC (13, 48, 49). Nevertheless, the use of SBC is associated with side effects such as local irritation, bleeding, and vasoconstriction (50, 51). In addition, a rapid increase in blood pressure may be seen in patients with stroke in the early stages immediately after the intranasal cooling application but not in comatose patients with cardiac arrest (52, 53).

Several studies have shown that the activity of tissue plasminogen activator (tPA) decreases as the temperature decreases (approximately 0.5% reduction of the lytic effect per 1°C decrease). This suggests that the activity of tPA depends on the temperature and may be affected by hypothermia treatment at the clot site (54–56). Therefore, this may be an important factor to consider when applying tPA along with SBC in acute settings.

It is important to note that the protective effects of hypothermia are not limited to the brain and have been demonstrated in other organ systems (57). The effects of SBC may vary depending on the type and level of injury, hospitalization period, cooling time, and rewarming rate. Although the therapeutic effects of SBC cannot last for more than a few hours or days, extending the cooling period beyond the acute phase might improve long-term outcomes (45, 58). Although experimental evidence is insufficient, protective effects on other organs for long-term SBC can be expected. Therefore, it might have been, so far, preferred to apply systemic cooling in the case of pan-ischemia, such as cardiac arrest. In cases of isolated brain ischemia, however, neuroprotective effects of hypothermia do not require whole-body cooling, and SBC thus represents an attractive alternative in cases of ischemic stroke (59).

## Methodological Descriptions of Selective Brain Cooling

**Figure 3:**
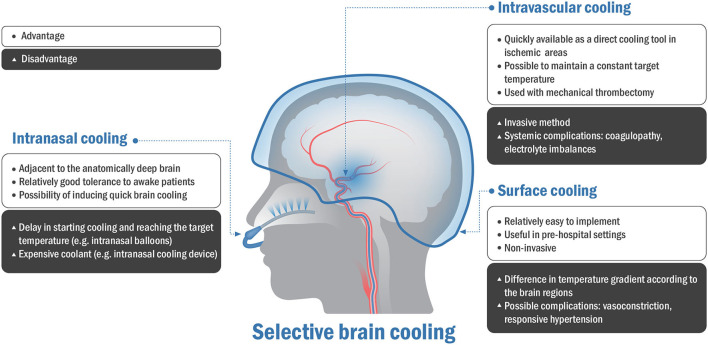
Advantages and disadvantages of three different methods of selective brain cooling.

The SBC can be performed in three approaches to reach the target temperature in patients with acute brain injury. The three main mechanisms of SBC are as follows: (1) direct surface cooling of the scalp, (2) intravascular cooling that enables heat exchange between the internal carotid artery and intracranial venous drainage, and (3) intranasal cooling that enables rapid heat exchange in the upper airway (12). Table 1 shows animal and clinical studies on different methods of SBC. Figure 3 shows the advantages and disadvantages of three different methods of the SBC.

**Table 1:** Animal and clinical studies on different methods of selective brain cooling.

**Reference**	**Patients/animals (n)**	**Population**	**Cooling methods**	**Results**	**Adverse events (Number of events/Number of cooling subjects)**
**Surface cooling**					
Battin et al. (60)	Humans (*n* = 26)	Birth-asphyxiated term newborn infants	Head cap (circulating water)	Selective brain cooling is a stable and reducing cerebral temperature	Death (1/13), seizures (9/13), pulmonary hypertension (3/13), decreased blood pressure (6/13)
Wang et al. (61)	Humans (*n* = 14)	Severe stroke or head injury	Cooling helmet	Rapid reduction of brain temperature from baseline and maintenance of temperature	None
Gluckman et al. (62)	Humans (*n* = 234)	Neonates with hypoxic-ischemic encephalopathy	Cooling caps	Improved survival without severe neurodevelopmental disability in infants with less-severe aEEG changes	Severe hypotension (3/112), unanticipated serious adverse event (1/112)
Poli et al. (63)	Humans (*n* = 11)	Severe ischemic or hemorrhagic stroke	Head and neck cooling device	Reduced brain temperature compared with baseline with a maximum of −0.36°C after 49 min	Severe hypertension (3/11), ICP crisis (3/11)
Zhao et al. (13)	C57BL/6J mice (*n* = 24)	tMCAO (60min)	Cooling pad (dry ice in an insulated storage container)	Reduced mortality from 31.8% to 0% and improved neurological outcomes for at least 35 days post-injury	None
**Intravascular cooling**					
Choi et al. (64)	Humans (*n* = 18)	Previous treatment of vascular malformations	Isotonic saline (into internal carotid artery)	Rapid reduction of brain temperature	None
Chen et al. (65)	Humans (*n* = 26)	Acute ischemic stroke	Cold isotonic saline	Reduced temperature by at least 2°C during infusion of the cold solution, and mild reduction in systemic temperature (maximum 0.3°C)	None
Wu et al. (66)	Humans (*n* = 113)	Acute ischemic stroke with mechanical thrombectomy	Intra-arterial selective cooling infusion (IA-SCI)	IA-SCI is associated with a reduction of final infarct volume and good safety profiles	Symptomatic intracranial hemorrhage (3/45), any intracerebral hemorrhage (16/45), all-cause death (9/45), coagulation abnormalities (1/45), pneumonia (14/45)
Wang et al. (67)	SD rats (*n* = 18)	tMCAO	Intra-carotid cold infusion	Ischemic striatal temperature is decreased by 2.3 ± 0.3°C within 2 min	None
**Intranasal cooling**					
Wang et al. (68)	Pigs (*n* = 16)	Cardiac arrest (VF)	Intranasal cooling device	Higher survival rate than in the control group	None
Castrén et al. (53)	Humans (*n* = 194)	Cardiac arrest patients	Intranasal cooling device	Brain cooling is faster, but there is no significant difference with the result of the original method	Periorbital emphysema (1/93), epistaxis (3/93), perioral bleed (1/93), nasal discolorations (13/93)
Poli et al. (69)	Humans (*n* = 20)	Intubated stroke patients	Cold infusions (CI) or nasopharyngeal cooling (NPC)	Brain cooling is faster during CI than during NPC	Systolic arterial pressure (2/10), shivering (1/10) in CI; systolic arterial pressure (3/10), shivering (1/10) in NPC
Nordberg et al. (47)	Humans (*n* = 677)	Cardiac arrest patients	Nasal catheters (delivery of a mixture of air or oxygen and a liquid coolant)	No significant improvement in survival but with better neurologic outcomes than usual care	Severe nosebleed (4/343), pneumocephalus (1/343), other adverse events (170/337)

*ICP, intracranial pressure; aEEG, amplitude-inetgrated electroencephalogram; tMCAO, transient middle cerebral artery occlusion; HTN, hypertension; SD, Sprague-Dawley*.

### Surface Cooling

In theory, local surface cooling through caps, helmets, and neckbands can improve neurological outcomes. Although brain temperature control mechanisms in humans are not clear, modulation of brain temperature relies on a complex interaction between the superficial (face and upper airways) and deep vascular beds, wherein cooling both surfaces (heat loss through the skull and venous sinuses) and upper airways (mucosa) contributes to heat exchange (61, 70). Particularly, multicenter randomized clinical trials have shown the feasibility of SBC immediately after birth to reduce neurodevelopmental sequelae of neonatal encephalopathy (62). Although the surface cooling method could be noninvasive and easy to apply in prehospital clinical environments, a significant reduction in core brain temperature comparing skin temperature, has not been confirmed in human (13, 63, 71).

Surface cooling with a helmet predominantly cools the superficial brain areas. It is therefore plausible that surface cooling may be more appropriate for cortical injuries, whereas systemic hypothermia may provide better neuroprotection in deep brain injuries (61, 71–73). However, these local irritations, in a study of healthy participants, combined with head and neck cooling also caused peripheral vasoconstriction and a significant increase in blood pressure by 15.3 ± 20.8 mmHg, while the heart rate was decreased by 6.5 beats per min (71). Such physiological responses are similar to those elicited by the cold face test, in which cold stimulation of the face induces peripheral sympathetic activation and simultaneous vagal activation with subsequent HR slowing (74).

Isolated neck cooling may cause less discomfort than combined cooling through the neck and the head (75). Neck cooling can have a greater impact on blood vessels and soft tissues and can be more useful than head cooling alone as it is less influenced by the bony skull used as insulation in head cooling (75). Given that head cooling alone requires cold conduction from the scalp to the brain, there may be some limitations in cooling the brain due to the thermal barriers. Previous studies have shown that head-neck cooling did not achieve selective brain cooling. Nevertheless, the lower target of neck cooling temperature could be attributed to successful brain cooling (63).

### Intravascular Brain Cooling

Endovascular cooling has been proven to be effective as a good neuroprotective method in many clinical conditions. The advantage of intravascular cooling is the direct cooling of the ischemic region and can be performed simultaneously with thrombectomy. It induces TH with the advantage of maintaining a constant target temperature during the maintenance stage and accurately controlling the heating rate during the rewarming stage (38, 64, 76). However, endovascular brain cooling is invasive and theoretically might be associated with procedural complications like arterial dissection (77). Among all techniques, cold saline infusion, which involves infusion of 0.9% ice-cold saline into the internal carotid artery at a rate of 30 mL/min for 10 min, has been proposed as a practical and effective cooling method by both human research and animal experiments (65, 77, 78). Cold saline infusion involved is safe for patients with acute ischemic stroke. It decreases ischemic cerebral tissue and core body temperatures by at least 2°C and 0.3°C, respectively (65). Local hypothermia caused by cold saline infusion helps reduce the infarct size and is more effective at the start of cooling prior to vessel recanalization (66, 67).

SBC can also be achieved through intra-arterial infusions *via* a micro-catheter during mechanical thrombectomy (46). A rodent stroke model showed that SBC successfully alleviated ischemic-reperfusion damage and improved functional outcomes. In cold saline infusion (i.e., 2.0 mL/min, approximately 50% of the physiological blood flow in the common carotid artery of rats) before, during, and after reperfusion, ipsilateral brain temperature was lower by 5.3 ± 1.8°C within an average of >42 s after flushing and injection than that at baseline (67). As a direct cooling modality in ischemia-related areas, this type of method has the advantage of selective and rapid cooling of the brain (79). We believe that some of the beneficial effects of this method could be due to mechanical flushing, increasing vessel patency and promoting vasorelaxation and hemodilution. This protocol also showed advantages in monkey stroke models and patients with stroke (80). Interestingly, several animal studies have reported that the combination of cold saline and magnesium sulfate (acting as an *N*-methyl-d-aspartate receptor antagonist that prevents excitotoxic cell damage) or low-dose albumin (improving microcirculation with multiple neuroprotective properties) has greater neuroprotective advantages than cold saline alone (81). The safety and validity of this method need to be verified in future randomized clinical trials (76, 79).

However, brain cooling rates and durations depend on the amount of cold salt water (0.9% saline) injected. A high load of saline can cause side effects such as abnormalities in hemodynamic variables and imbalance in serum electrolytes (65, 81). Given that most patients who undergo TH have severe inflammation and immobility, they tend to be at a high risk of venous thrombosis. Catheter-induced thrombosis (CRT) causes complications in 2–67% of cases, and the use of endovascular catheters in TH may further increase the risk of catheter-induced thrombosis due to local and systemic effects (82). Thus, catheter-induced thrombosis can be an important limitation of this promising technique.

### Intranasal Cooling

Focal TH through the nasopharyngeal structure may be the most efficient approach for SBC (83). First, blood flow to the brain flows from the internal carotid artery to the cavernous sinus; contacts the deep part of the brain far from the scalp, which physiologically acts as a brain temperature insulator; and is anatomically close to the nasopharynx (12). Second, thermal conductivity through the base of the skull can also be effective because colling-induced heat exchange in the skull is the result of direct heat loss to the air and evaporation of water. This heat loss can account for up to 10% of the total body heat loss under normal conditions. In the transnasal approach, the net cooling effect is determined by airflow rate, humidity, and air temperature (84).

Transnasal high flow of dry air safely induced and maintained either normothermia (48) or hypothermia (49, 68, 85) at the brain temperature in preclinical models and preliminary clinical data. Intranasal balloons circulated with cold saline safely provided brain temperature reduction (−1.7 ± 0.8°C after 60 min of intranasal cooling) and are well tolerated in awake patients (86). Esophageal cooling devices circulating water at adjustable temperatures have been shown to induce and accurately control core temperature without major adverse events in cancer survivors (47, 87). Nevertheless, these devices are associated with a long delay in cooling initiation and reaching the target temperature. Thus, its use in combination with other strategies using intravenous ice-cold fluids or surface brain cooling may be more favorable (87, 88).

The intranasal cooling device vaporizes perfluorocarbon along with oxygen at a flow rate of 40 to 60 L/min using a catheter system into the nasal cavity, leading to a fast induction of hypothermia (15). Therapeutic hypothermia increases survival and neurological outcomes after out-of-hospital cardiac arrest. This non-invasive cooling method can be performed intra-arrest and provides continuous cooling, primarily of the brain (47).

However, although the administration of oxygen-containing perfluorocarbon gas and compressed air into the nasopharynx can cool the brain, this method does not guarantee accurate temperature control and still has safety issues with respect to localized overcooling and tissue damage in the nose and the cheeks due to freezing (15, 89). In addition, because neuroprotection against brain damage may require at least 12–24 h of brain cooling, the cost of coolant can be high if an intranasal cooling device is used throughout the treatment period (15).

SBC delivered to the nose and the esophagus using circulating cold saline (1–3 L/min) is one of the most promising approaches, achieving effective heat exchange with the nasopharyngeal surface and having a good safety profile (30, 47, 69). However, such a transnasal cooling technique has not yet been proven to achieve SBC without lowering the body temperature. Further, the side effects related to the decline in body temperature are yet to be clarified (90). Studies on intranasal evaporation cooling by the intranasal cooling device in patients with post-cardiac arrest syndrome have shown that it is a safe and feasible modality (53, 91). In the Pre-Return of Spontaneous Circulation (ROSC) IntraNasal Cooling Effectiveness trial, the device decreased the tympanic temperature (a surrogate measure of brain temperature) to 34.2°C within an average of 34 min unlike whole-body cooling, which has an induction time of 1–7.4 h (53). A recent stroke trial also confirmed the efficacy of the device in the abovementioned trial, with the brain temperature decreasing by 1.2°C within an average of 58 min after the device application. Meanwhile, there have been reports of patients showing a significant increase in systolic arterial pressure without the increased intracranial pressure for several minutes at the start of the cooling (52). In this regard, individual assessment of efficacy and safety issues may be required for patients who are expected to be affected by initial transient hypertension, such as hemorrhagic stroke or post-ROSC patient and for those who are not.

## Future Directions and Studies in Selective Brain Cooling Based on Coping Mechanisms Observed in Animals

Various SBC strategies are used to lower brain temperature, but it is a natural phenomenon observed in animals. In this section, various innate cooling methods in animals are examined, and the feasibility of applying these methods in the future clinical setting is discussed.

### Evaporative Cooling

Animals living in hot deserts survive by lowering their body temperature through various methods, with most mammals using evaporative cooling methods to cope with high body temperatures (92). The mechanism of evaporative cooling varies from species to species, but it mainly includes gasping, sweating, and drooling (93). In general, larger animals (e.g., kangaroos, elephants, and camels) rely more on sweating, while smaller animals (e.g., red hartebeest) rely on gasping (94).

Heat stress occurs in extremely hot environments, resulting in an imbalance between internal demands and the environment, where the capacity to dissipate heat is altered (95). During heat stress, physiological mechanisms promote heat loss through cutaneous vasodilation and sensible heat loss by conduction, convection, and radiation due to the thermal gradient between the animal and the environment (96). Sweating and gasping are essential heat adaptation mechanisms for the Sphynx cat, the Mexican hairless dog, and the Chinese Crested dog. Kangaroos also have a unique cooling system comprising a network of hundreds of small blood vessels immediately below the forearm surface (97). They lick their arms to cool down, evaporate moisture in their skin, and lower their body temperature. Rolling in mud also provides a means of skin cooling as the water evaporates; this is very useful for elephants or pigs that are particularly exposed to high temperatures and dispel only a small percentage of their body heat by evaporation without sweating (98).

Heat dissipation *via* evaporation can be an important therapeutic strategy to control body temperature under various heat loads. Using a coolant such as spraying water may be the easiest method for applying evaporation in human.

### Heat Dissipating With Carotid Rete

The brain is an essential organ that is remarkably vulnerable to high temperatures. Therefore, some herbivorous mammals such as sheep, goats and antelopes, and gazelles use a counter-current heat-dissipating network known as the carotid rete or rete mirabile to keep the brain cooler than the body (99–101). The carotid rete is a functional structure that allows brain cooling in herbivorous mammals, enabling them to continuously avoid large predators (102). The rete is a web structure of arteries and veins within the sinus at the base of the brain (102). Warm blood flowing into the brain moves from the carotid artery to this web of small arteries and transfers part of the heat to cooler venous blood flowing in the opposite direction from the nasal passages (103). Consequently, the cooled arterial blood travels toward the brain, passing through this structure, thus acting like a radiator (102). Therefore, the carotid rete should be referenced as a biological prototype when developing new SBC devices in human.

### Hibernation

Hibernation allows animals to survive under hypothermia and hypometabolism, adapting to cold temperatures and reduced feeding (104). Animal hibernation is characterized by reduced metabolism, severely decreased heart and respiratory rates, and marked lowering of body temperatures to a level few degrees higher than ambient temperature. Therefore, hibernation can lead to differences in brain and body temperatures. In particular, the brain temperature decreases to 2–3°C lower than the ambient temperature. The differences in body and brain temperatures help in the uncomplicated survival of hibernating animals (105). Brown adipose tissue (BAT) is a unique heat-generating tissue of mammals that quickly produces heat through non-shivering thermogenesis (106). Lipid catabolism occurs due to BAT in natural hibernation, and BAT mainly increase in autumn. Lipids stored at certain points in hibernation are consumed at high speeds by BAT, which creates heat and arousal in hypothermic hibernation (107, 108). Non-shivering thermogenesis from the BAT is induced through activation of the mitochondrial uncoupling protein (UCP1) (109). UCP1 is expressed differently according to the tissue during hibernation (110). Significant UCP1 expression was observed in the nervous tissue of carp after cold exposure, suggesting that there may be a neuroprotection mechanism through local thermoregulation.

Therefore, artificial hibernation can be feasible for non-hibernating species with a difference between brain and body temperature such as humans. In addition, an SBC strategy using artificial hibernation materials and devices could be feasible for irreversible brain damage due to blood flow arrest.

### Selective Brain Cooling With Yawning

Yawns were recently proposed as a cooling mechanism of the brain in mammals including humans (111). Three factors affect brain temperature:(1) rate of arterial blood flow, (2) temperature of arterial blood, and (3) amount of metabolic heat production (112). Yawning can change the first two variables, causing significant changes in the circulatory system, including accelerating the heart rate by up to 10 additional beats per minute (111, 113). When yawning, the jaw is opened widely, and the pressure to breathe deeply cools the brain by allowing warm blood to escape from the skull. The mechanism by which yawning cools the brain involves the change in the ventilation rates associated with an increase in facial blood circulation (114, 115). Animals that regulate temperature through yawning include birds, mice, humans, and birds. One study found a difference in facial temperature between birds that did and those that did not yawn (116).

**Figure 4:**
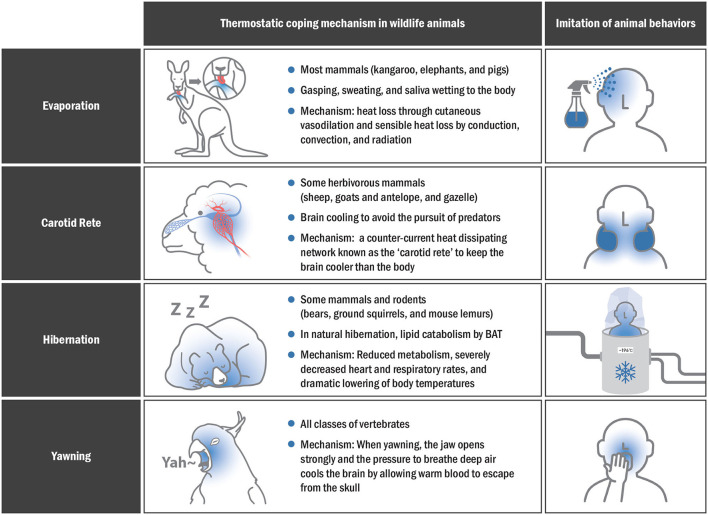
Thermostatic mechanisms in animals and imitation of their behaviors for effective SBC application in humans.

In human, those with the highest facial temperature were found to have yawned faster and more frequently (117). A recent study reported that the change in facial temperature in high-yawning rats is closely similar to the pattern of decreased facial temperature in avian species with more yawns (118). Newborns experience an average temperature decrease of −0.36°C in the cerebral and eye region 10–20 s after yawning, which is consistent with the results of a decrease in facial temperatures in the high-yawning rats (115, 119). Given the brain cooling mechanism in numerous yawning studies, pharyngeal cooling could be a feasible strategy for rapid SBC. Figure 4 shows various SBC-related surviving skills in animals that could be applied to humans.

## Conclusion

TH, which prevents irreversible neuronal necrosis and ischemic brain damage, is an effective modality for post-cardiac arrest syndrome and neonatal encephalopathy. The current literature supports the strong potential of applying animal SBC strategies for neuroprotection in human with respect to their physiological mechanisms and the absence of serious systemic adverse events.

## Author Contributions

JH participated in the conception of this review, the interpretation of data, writing the manuscript, and revising it critically for important intellectual content. EC participated in the interpretation of data, drafting the article, and revising the manuscript. SP participated in the acquisition of data. All authors contributed to the article and approved the submitted version.

## Funding

This work was supported by a grant (2021-DD-RD-0047) from the Korea Innovation Foundation (INNOPOLIS) from the Ministry of Science and ICT, Republic of Korea.

## Conflict of Interest

The authors declare that the research was conducted in the absence of any commercial or financial relationships that could be construed as a potential conflict of interest.

## Publisher's Note

All claims expressed in this article are solely those of the authors and do not necessarily represent those of their affiliated organizations, or those of the publisher, the editors and the reviewers. Any product that may be evaluated in this article, or claim that may be made by its manufacturer, is not guaranteed or endorsed by the publisher.

## Abbreviations

TH: therapeutic hypothermia
SBC: selective brain cooling
TTM: targeted temperature management
BAT: brown adipose tissue.
